# Thermal tomography for monitoring tumor response to neoadjuvant chemotherapy in women with locally advanced breast cancer

**DOI:** 10.18632/oncotarget.16569

**Published:** 2017-03-25

**Authors:** Qi Wu, Juanjuan Li, Si Sun, Xiaoli Yao, Shan Zhu, Juan Wu, Qian Liu, Xiaojun Ding, Manman Shi, Kaiyang Li, Shengrong Sun

**Affiliations:** ^1^ Department of Breast and Thyroid Surgery, Renmin Hospital of Wuhan University, Wuhan, Hubei, P. R. China; ^2^ Department of Clinical Laboratory, Renmin Hospital of Wuhan University, Wuhan, Hubei, P. R. China; ^3^ Department of Pathology, Renmin Hospital of Wuhan University, Wuhan, Hubei, P. R. China; ^4^ Department of Electronic Science and Technology, School of Physics and Technology, Wuhan University, Wuhan, Hubei, P. R. China

**Keywords:** thermal tomography, neoadjuvant chemotherapy, breast cancer

## Abstract

**Background & Aims:**

This study aims to analyze the feasibility and predictive value of thermal tomography (TT) for monitoring early treatment response in patients with locally advanced breast cancer (LABC) receiving neoadjuvant chemotherapy (NAC).

**Methods:**

Patients with LABC who were due to receive six cycles of NAC were examined by TT prior to NAC, the second cycle of NAC, the fourth cycle of NAC and surgery. Changes in TT parameters and ultrasonography were correlated with pathologic response to NAC, and the predictive value was assessed.

**Results:**

Forty-four patients were evaluable for response (25 pathologic responders and 19 nonresponders). As early as after the first cycle of NAC, changes in the TT parameters ΔTs, ΔTn, and ΔTa correlated significantly with pathologic response (*P* < 0.05). The best predictor of pathologic response after the 6th cycle of NAC was TT (area under the receiver operating characteristic curve, 0.794), as opposed to cross-sectional areas and the longest diameter by ultrasonography.

**Conclusions:**

TT allows for monitoring early tumor response to NAC and can predict pathologic response in the early stages of therapy. Therefore, TT could be used as a novel imaging modality to monitor NAC treatment.

## INTRODUCTION

Neoadjuvant chemotherapy (NAC) was originally applied in locally advanced inoperable breast cancer to enable surgical resection. Subsequently, a novel use for NAC has emerged: it is utilized to shrink tumors and facilitate breast-conserving surgery in operable breast cancer [1, 2]. Moreover, pathologic complete response (pCR) to NAC has consistently been associated with increased long-term survival [1, 3]. Although NAC is a crucial treatment strategy for locally advanced breast cancer (LABC), a considerable number of patients are nonresponsive to NAC or even experience tumor progression while receiving NAC [4, 5]. Thus, the development of new monitoring methods is essential for improving the course of therapy.

Currently, the main methods used to monitor tumor response to NAC rely on integrating clinical assessment and breast imaging modalities, such as positron emission tomography (PET), ultrasonography, or magnetic resonance imaging (MRI) [6, 7]. However, evaluation of tumor regression by clinical assessment is subjective and correlates poorly with treatment response [3]. Similarly, ultrasonography measures the extent of tumor size reduction at only a two-dimensional level, and the accuracy of this method has been disappointing. Furthermore, both PET and MRI are associated with considerable cost and inconvenience, and the correlation between imaging techniques and pCR assessment is limited [8, 9].

Thermal tomography (TT) is a novel, noninvasive imaging technique that has been used for early detection of breast tumors [10–12]. TT employs infrared radiation to obtain three-dimensional (3-D) heat distribution information, and these thermal properties of cells and tissues are strongly connected to pathological changes [13, 14]. Specifically, metabolism is accelerated in tumor cells, which increases blood flow and cell growth [15], and tumor can be partially attributed to angiectasis and angiogenesis [16–19]. These changes all increase the temperature of the tumor. In untreated tumor tissues, the parameters of TT, such as the surface temperature and q-r curve, are much higher than in benign tissues. Moreover, these differences are attenuated by effective therapy, which inhibits metabolism and blood flow in the tumor, and liquefaction and necrosis at the center of advanced malignant tumors lowers the temperature in this region [20, 21]. TT not only detects temperature changes but also generates a q-r curve to evaluate the temperature distribution characteristics of a tumor at different stages, when the heat intensity varies with the depth of tomography. In addition, because TT is noninvasive and does not require contrast agents, it is a promising modality for frequent measurements of tumor response to NAC.

In the present study, we used TT to evaluate women receiving NAC for the treatment of LABC at regular intervals throughout the course of therapy. This study aimed to analyze the feasibility and predictive value of TT for monitoring treatment response in patients with LABC receiving NAC. Moreover, this study compares TT with ultrasonography for assessing tumor response.

## RESULTS

### Patient and tumor characteristics

The study consisted of 44 females, 25 responding and 19 nonresponding patients, with an average age of 47.30±10.63 years (range 26-64) for whom complete information was available. Of the 44 breast lesions, only 1 patient achieved pCR after six cycles of NAC, and none of the patients experienced PD according to pathologic response (Miller and Payne criteria) measurements. As shown in Table 1, the majority of malignancies were invasive ductal carcinoma. The median tumor longest diameter was 4.03±1.414 cm (responding: 3.74±1.191 cm, nonresponding: 4.41±1.617 cm), and the median cross-sectional tumor area was 10.63±8.94 cm2 (responding: 8.14±5.26 cm2, nonresponding: 13.91±11.59 cm2; *P* < 0.05). Other characteristics, such as menopausal, ER, and PR status, did not significantly differ between the two groups. The distribution of cancer-associated characteristics, such as breast subtype, was classified following a clinical assessment and the REC of response measurements after six cycles, as shown in Supplementary Figure 1.

**Table 1:** Patient and tumor characteristics

Characteristic	All patients (*N* = 44)	Response (*N* = 25)	Nonresponse (*N* = 19)
**Age in years (median; range)**	47.30±10.63	47.28±10.15	47.32±9.89
** Menopausal status**			
** Premenopausal**	25	17	8
** Postmenopausal**	16	8	8
** Unknown**	3	0	3
**Tumor histology**			
** Invasive ductal carcinoma**	40	24	16
** Invasive lobular carcinoma**	2	0	2
** Others**	2	1	1
**Size in cm^2^ (median; range)**	10.63±8.94	8.14±5.26	13.91±11.59*
**Longest diameter in cm (median; range)**	4.03±1.414	3.74±1.191	4.41±1.617
**Stage primary tumor^1^**			
** T1C**	1	1	0
** T2**	33	19	14
** T3**	10	5	5
**Grade**			
** II**	31	20	11
** III**	13	5	8
**ER**			
** Positive**	21	12	9
** Negative**	23	13	10
**PR**			
** Positive**	29	17	12
** Negative**	15	8	7
**Her2**			
** Positive**	22	12	10
** Negative**	22	13	9
**Ki-67**			
** ≤ 14**	14	7	7
** > 14**	30	18	12
**Breast subtype**			
** HR+/HER2-**	11	4	7
** HR+/HER2+**	12	9	3
** HR-/HER2+**	10	4	6
** TN**	11	8	3

NOTE: ^1^Staging according to American Joint Committee on Cancer guidelines. * Two-tailed t-test *P* values estimate significance of the differences between the two groups (*P* < 0.05). HR: hormone receptor, HER2: human epidermal growth factor receptor 2, TN: triple negative. The measurement data were expressed as M ± SD.

### Comparison between response and nonresponse according to ultrasonography and thermal tomography

A total of 44 patients received ultrasonography and thermal tomography as required. The mean ΔTs for tumors at baseline was 2.58°C for the responding group and 3.10°C for the nonresponding group. Similarly, the mean ΔTn for tumors at baseline was 2.40°C for the responding group and 3.25°C for the nonresponding group, and the ΔTa for tumors at baseline was 1.30°C for the responding group and 1.80°C for the nonresponding group. These differences were not significant. According to the characteristics of the q-r curve, blood vessels and isothermals, the images of tumors were characteristic of malignancy.

**Figure 1:**
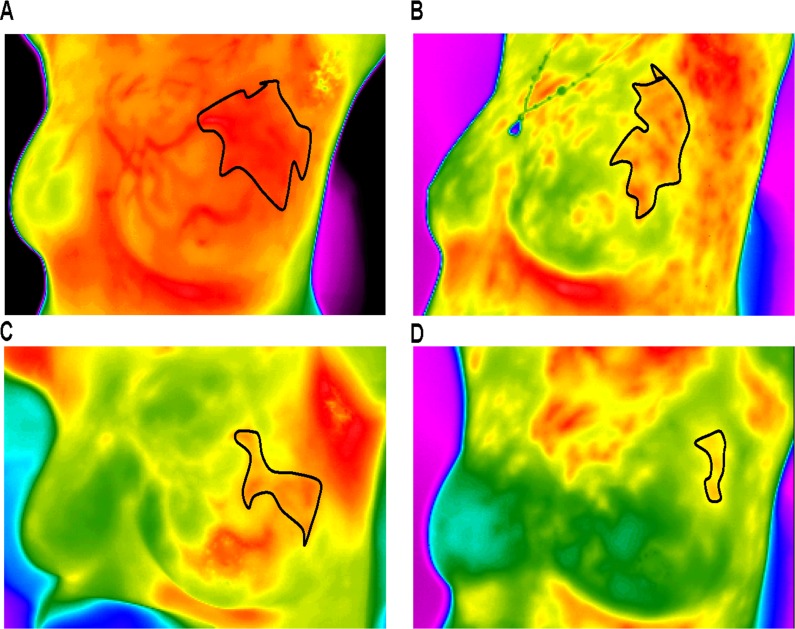
Thermal tomography images of a typical response before and after NAC **A.**-**D.** show a significant decrease in surface temperature in the area of the tumor before and after one, three, and six cycles of NAC (these measurements can vary from black (coolest) to white (warmest), with 14 “intermediate” colors).

**Figure 2:**
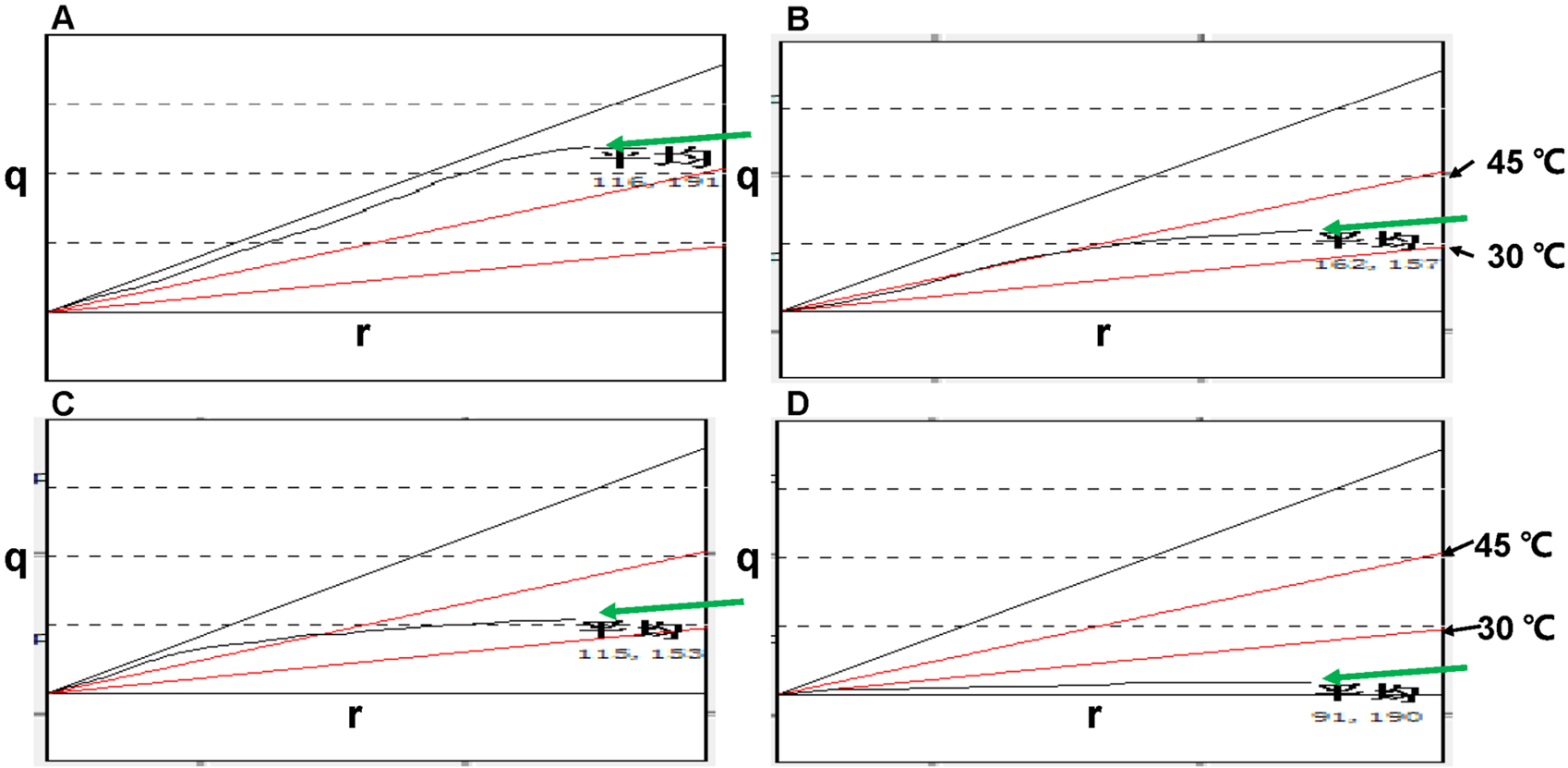
q-r curves of a typical response before and after NAC **A.**-**D.** show a significant decline in q-r values before and after one, three, and six cycles of NAC (the green arrows show q-r curves; the black arrows show upper and lower limits; the horizontal axis represents the depth r; the vertical axis represents the quantity of heat q).

**Figure 3:**
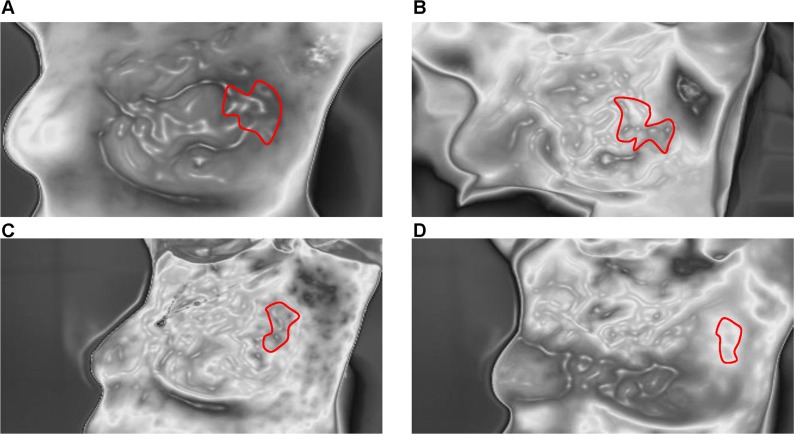
Blood vessel scans of a typical response before and after NAC **A.**-**D.** show significant shrinkage and discontinuity of blood vessels before and after one, three, and six cycles of NAC.

**Figure 4:**
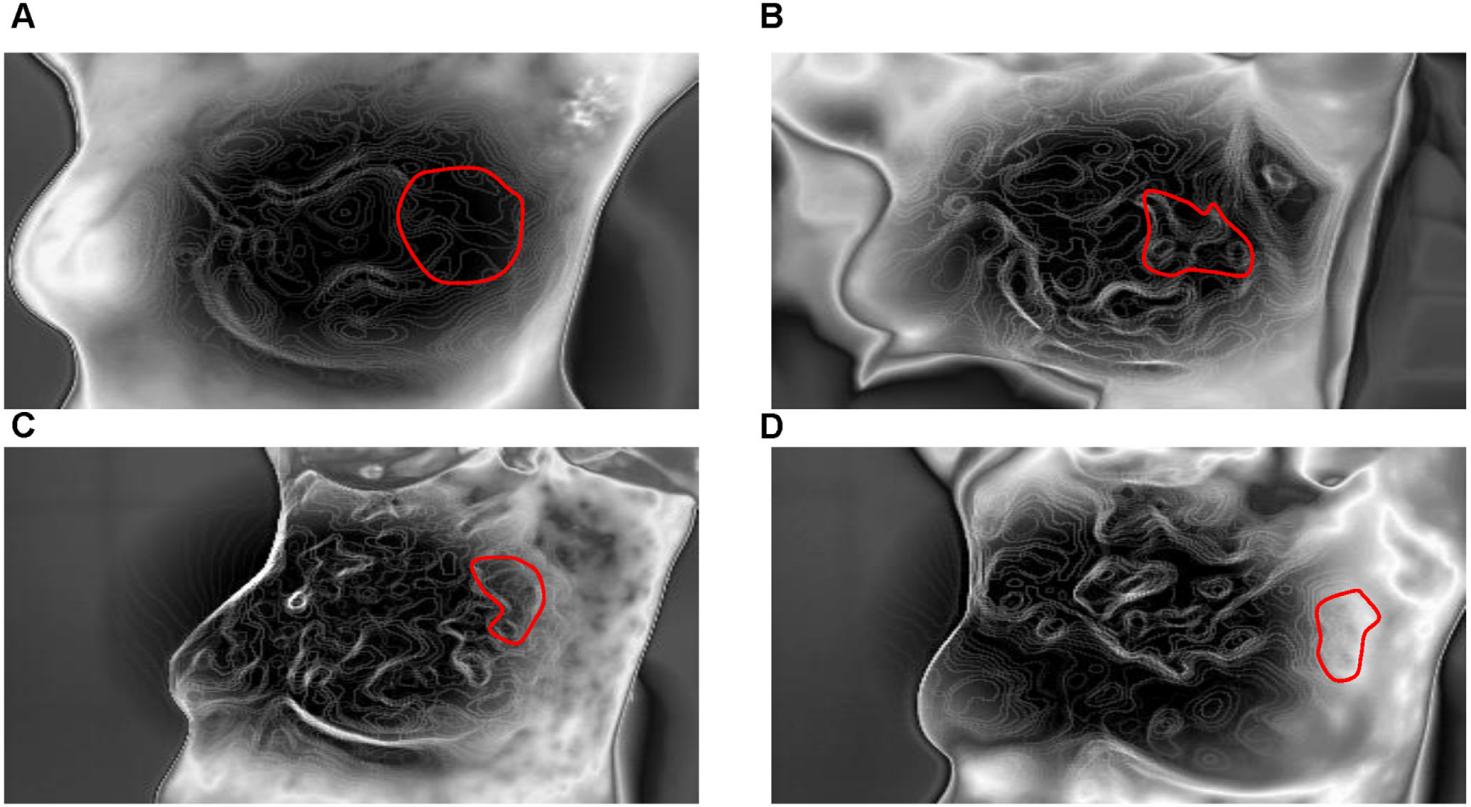
Isothermal images of a typical response before and after NAC **A.**-**D.** show significant sparsity of isothermals before and after one, three, and six cycles of NAC.

Furthermore, the values relative to baseline were adopted to intuitively assess trends. However, based on pathologic criteria, the relative ΔTs, ΔTn and ΔTa values shown in Table 2 significantly differed between the responding and nonresponding groups after 1 cycle of NAC (*P* < 0.05). The q-r curve relative to baseline also indicated a significant decrease. Due to their subjective nature, it is difficult to evaluate diagnosis based on blood vessels and isothermals, though changes can be assessed from TT images. Figures 1, 2, 3, 4 show representative TT images of the response group during NAC therapy (TT images are shown in Figure 1A-1D; q-r curves are shown in Figure 2A-2D; blood vessel scans are shown in Figure 3A-3D; and isothermals are shown in Figure 4A-4D). After three and six cycles, the ultrasonography measurements of cross-sectional areas significantly differed between the responding and nonresponding groups (*P* < 0.05; Table 2). In addition, the sizes of the tumors on scans decreased after NAC in both groups.

**Table 2:** Differentiation between response and nonresponse using thermal tomography and ultrasound

	Baseline	1 cycle	3 cycle	6 cycle
**ΔTs**				
** Response (%)**	2.58±1.38 (100)	1.93±0.61(84.2)	0.90±0.25 (46.8)	0.58±1.89 (27.8)
** Nonresponse (%)**	3.10±1.13 (100)	5.55±3.18^*^ (211.9)	2.00±0.42 (66.4)	1.45±0.70* (50.6)
**ΔTn**				
** Response (%)**	2.40±1.12 (100)	1.53±1.38 (74.3)	0.50±0.25 (30.5)	0.30±0.22 (16.7)
** Nonresponse (%)**	3.25±0.92	6.00±3.96* (210.3)	1.90±0.42 (59.0)	1.80±0.57* (60.3)
**ΔTa**				
** Response (%)**	1.30±0.67 (100)	0.73±0.17 (70.4)	0.25±0.07 (30.6)	0.35±0.58 (36.2)
** Nonresponse (%)**	1.80±0.14 (100)	1.75±1.06* (99.8)	0.65±0.13 (36.1)	0.40±0.14 (22.0)
**q-r curve**				
** Response (%)**	100	75	50	25
** Nonresponse (%)**	100	100*	100*	100*
**Size in cm^2^**				
** Response (%)**	8.14±5.26 (100)	--	5.61±4.52 (41.3)	2.34±1.44 (20.1)
** Nonresponse (%)**	13.91±11.59 (100)	--	8.55±10.80^*^ (84.5)	8.25±10.49* (79.6)

NOTE: ΔTs: tumor surface temperature difference between neoplastic side and healthy one, ΔTn: nipple temperature difference between neoplastic side and healthy one; ΔTa: average temperature difference between neoplastic side and healthy one; Size: the measurements of cross sectional area by ultrasound. The measurement data were expressed as M ± SD. The value in “()” represents the relative content to baseline. *The Mann–Whitney *U*-test *P* values estimate significance of the differences between the two groups (*P* < 0.05).

### Predictive value of ultrasonography and thermal tomography

**Figure 5:**
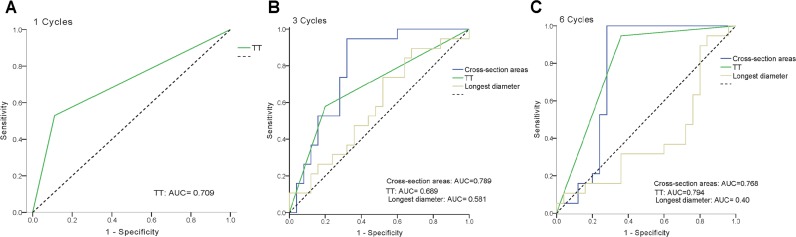
Predictive value of thermal tomography (TT) and ROC curves for TT and conventional modalities by ultrasonography using the longest diameter (REC) and cross-section area measurement (SM) after one, three, and six cycles of NAC The dashed diagonal shows an ROC for a completely random classification result with an AUC of 0.5.

The ROC curves shown in Figure 5 indicate the accuracy of prediction for different trade-offs based on TT, REC and SM values, which were normalized to baseline values. After considering all parameters, specialists assessed and divided the cohort into responding and nonresponding groups based on TT. ROC curves showed that TT and the normalized SM had comparable predictive values and were more accurate than REC. Based on the AUC, the predictive power for TT was similar for all time points, with AUC values of 0.841, 0.689, and 0.794 after one, three, and six cycles, respectively. The SM AUC was 0.789 after three cycles and 0.768 after six cycles. However, the REC AUC was 0.581 after three cycles and only 0.40 after six cycles.

## DISCUSSION

Given the increasing use of NAC treatment, innovative imaging modalities that can improve the early predictive value of tumor response are needed to identify patients who may benefit from treatment. Specifically, early detection of nonresponding tumors could prevent unnecessary NAC treatment or help select suitable NAC regimens. The current study demonstrated the clinical use of TT for monitoring treatment response to NAC in patients with LABC. TT monitoring revealed a significant difference in the thermal parameters of tumors between responding and the nonresponding groups as early as after the first cycle of NAC. Moreover, the predictive value of TT for determining tumor response corresponded to that of ultrasonography SM after three cycles of NAC and was superior to the predictive value of REC.

TT revealed significant decreases in ΔTs, ΔTn, and ΔTa values of tumors that responded to NAC. Due to individual differences, the absolute quantities of ΔTs, ΔTn, and ΔTa varied greatly and may have been influenced by many factors, such as the environmental temperature. This large inter-individual variability obscured significant differences between the groups. Therefore, we regarded them as standardization with respect to baseline, directly reflecting the effects of NAC. Furthermore, the q-r curves showed decreases from 30-45°C, which represents a malignant tumor, to 15-30°C and even 0-15°C. As indicated in previous studies, malignant tumors are characterized by a temperature of 30-45°C, which is significantly higher than the temperature of benign tumors and normal tissues [12]. The shrinkage of high-temperature areas observed in this study suggested reductions in tumor size. However, the sectorial q-r curve method could not precisely reflect continuous change, which limited the predictive power for monitoring the response to NAC. This limitation remains to be overcome in future research.

Blood vessel scans are promising biomarkers for treatment response because tumor growth strongly depends on angiopoiesis, especially for larger tumors. Patients receiving NAC often harbor much larger tumors, and these tumors consequently contain more blood vessels with quicker blood flow to fulfill the demands of tumor growth. TT can clearly visualize the spread and morphology of blood vessels. In the present study, the blood vessels of malignant tumors were enlarged and centralized yet were reduced in size and became discontinuous in the responding group after three or six cycles of NAC. Corroborating our results, Mei-Lin et al. reported that changes in the micro-vessel functionality of breast cancer blood vessels, as depicted by dynamic contrast-enhanced magnetic resonance imaging (DCE-MRI), soon after starting anthracycline-based NAC can predict the final clinical and pathologic response [22]. Blood vessel subtypes of a tumor have been suggested to exhibit differential susceptibility to anti-cancer therapy [23]. Notably, TT blood vessel scans are noninvasive and do not require contrast agents. Thus, compared with MRI, TT can be used more frequently to measure tumor response to NAC.

Traditionally, tumor size measured by ultrasonography is an imperfect assessment of response to NAC because it can both over- and underestimate the histologically defined tumor size. However, in our study, normalized tumor cross-sectional areas (SM) decreased from the baseline values after six cycles of NAC. The predictive power of ultrasonography measurements after six cycles decreased because nonresponding tumors based on MP criteria showed no contrast enhancement after six cycles. Moreover, the inclusion of 11 HER2-/ER+ breast cancers affected the accuracy. As previously mentioned, MRI volume measurements were able to accurately assess response to NAC, and the AUC was 0.97 after three cycles and 0.71 after six cycles [24]. Indeed, MRI volume measurements have been identified as the most accurate assessment method, and MRI was found to be superior to ultrasonography in both the initial staging of primary breast cancer and the assessment of residual disease at the end of treatment [25–27]. However, this accuracy was reduced for HER2-/ER+ breast cancer [28]. Furthermore, Harriet and colleagues adopted diffusion-weighted MRI (DW-MRI) to predict treatment response because this technique is sensitive to microstructural changes at the cellular level during therapy at an earlier time point than anatomical changes. Their results demonstrated that the apparent diffusion coefficient (ADC) is a promising biomarker for predicting treatment response, and low pretreatment ADC values are often predictive of a better outcome. However, ultrasonography can only image a tumor in a two-dimensional horizontal plane and does not provide molecular imaging data, reducing its predictive value compared with that of MRI or DW-MRI.

This study has several limitations. TT could not accurately localize a tumor; therefore, localization was partly based on conventional ultrasonography and mammography data acquired before therapy. The inability of TT to localize the tumor may result in undervaluation of tumor changes and consequently decrease the precision of the technique. Moreover, TT blood vessel scans may be influenced by many factors, such as tumor size. However, neovascularization was minimal or absent in small tumors, and dysplastic blood vessels were difficult to observe. Thus, merging molecular biomarkers and TT may improve assessment and provide more functional parameters to further increase the accuracy of assessment. In addition, the relatively small number and heterogeneity of the patients included may have resulted in bias.

In conclusion, TT allows for monitoring early response of tumors to NAC and can distinguish responding tumors from nonresponding tumors during the early stages of therapy. Therefore, TT could be used as a novel imaging modality to monitor NAC treatment and assist in individualized patient care.

## MATERIALS AND METHODS

### Patients

From January 2014 to July 2016, patients received six cycles of NAC over three weeks consisting of TAC (docetaxel, doxorubicin, and cyclophosphamide) or EC-T (doxorubicin and cyclophosphamide in three cycles over three weeks and docetaxel in three cycles over three weeks). The method used to monitor tumor response to NAC depended on the clinical assessment before each cycle and three ultrasonography examinations (before NAC, before the fourth cycle, and before surgery). In addition, four examinations by TT were planned for each patient (before NAC, before the second cycle, before the fourth cycle, and before surgery). TT was executed using an available breast imaging system (applied by Prof. Kaiyang Li, Wuhan University, School of Physics and Technology, Department of Electronic Science and Technology), and TT theory has been described previously [10–12]. The inclusion criteria were patients with breast cancer with tumors larger than 2 cm or with lymph node metastasis, without distant metastasis, and those eligible for NAC treatment. Patients who had undergone previous breast surgery, radiation therapy or chemotherapy were excluded. All acquired data were anonymized, and the included patients provided informed consent. The study was conducted in accordance with the Declaration of Helsinki and with approval from the Ethics Committee of Renmin Hospital of Wuhan University (Wuhan, China).

### Ultrasonography assessment of tumor response to NAC

Tumor response to NAC was assessed by ultrasonography based on the response evaluation criterion of the longest diameter of the enhancing tumor target lesion (REC). A complete reduction of the target lesion was defined as complete response (CR). A reduction in tumor size by at least 30% was defined as partial response (PR). Stable disease (SD) was defined as an increase in tumor size less than 20% or a decrease less than 30%. Progressive disease (PD) was defined as an increase in tumor size of at least 20%. The tumor cross-sectional area on ultrasonography images was calculated using dedicated software. All measurements were performed by an experienced sonographer.

### Pathologic assessment of tumor response to NAC

Standard pathology analysis of excised tissues was used to assess subject outcomes. The excised specimens were fixed, embedded in paraffin as tissue blocks and then serially sectioned at a thickness of approximately 5 mm before being stained with hematoxylin and eosin (HE). The final pathology examinations were evaluated by an experienced pathologist for grading of pathologic tumor response of the primary breast lesion using Miller and Payne criteria (MP criteria) [29].

### Thermal tomography assessment of tumor response to NAC

The thermal tomography system was applied by Prof. Kaiyang Li (Wuhan University, School of Physics and Technology, Department of Electronic Science and Technology), as described previously [10, 12]. The main component of this system consists of a highly sensitive infrared imager and a computer processing system. The data processing computer, the movement of the infrared camera and the medical record printer are controlled using the control console. The infrared thermal image is processed and analyzed by the computer processing system. The room temperature was maintained at a uniform temperature of approximately 18.5°C. The temperature resolution of the uncooled focal plane infrared thermal imager was 0.08°C, the spatial resolution was 1.4 mrad, the pixel range was 320 × 240, and the focusing range was 0.5 m to ∞, which is appropriate for a tool measuring the temperature of the human body surface. The examining room contained a rotary round stool rotating 360 ° that was designed to ensure that the acquired image information from any body part be visualized precisely.

The parameters of TT were recorded, including the ΔTs (tumor surface temperature difference between neoplastic side and the healthy side), ΔTn (nipple temperature difference between the neoplastic side and the healthy side), ΔTa (average temperature difference between the neoplastic side and the healthy side), q-r curve, blood vessels and isothermals. The heat distribution in the human body and metabolism are reflected by the shape of the q-r curve. A flat surface was created to signify the distribution and trend of the q-r curve, and this surface was segmented into four sections with three straight lines, with included angles with the horizontal axis of 45°C, 30°C and 15°C. The q-r curve of most female breast tumors is at 30-45°C, whereas that of most benign tumors is at 15-30°C; the q-r curve lies at 0-15°C for most hyperplastic or normal tissues, as described previously. These colored images consisted of color pixels that each reflect a single temperature measurement. These measurements can vary from black (coolest) to white (warmest), with 14 “intermediate” colors [11]. The blood vessels of most malignant tumors tend to be enlarged and centralized. Similarly, the isothermals of most malignant tumors are dense and disorganized.

### Data and statistical analysis

The main goal of this study was to assess the feasibility of TT for the early assessment of tumor response to NAC based on pathologic response after the first cycle. All subjects were divided in two subgroups based on MP criteria: nonresponding (MP 1; no decrease in tumor cellularity after NAC) and responding (MP 2-5) groups. For TT, changes in all parameters relative to baseline were compared between the two groups and analyzed for significant differences using the Mann-Whitney U test. The classifier was evaluated by estimating the ROC. The final predictive value of the evaluation was calculated based on the average ROC. All statistical analyses and all charts were generated using SPSS 19.0 (IBM Corporation, Armonk, NY, USA). A two-sided *P* value < 0.05 was considered to indicate a significant difference.

Compared with REC, the sensitivity and specificity of both TT and SM for predicting tumor response to NAC were assessed. The assessment based on TT criteria was numerically ordered as follows: nonresponding - 0 and responding - 1. The REC and SM after three and six cycles were normalized to the respective baseline values. The subjects were divided into two groups based on MP criteria, nonresponding and responding, and normalized cross-sectional areas after three and six cycles were compared between the groups. The significance of these differences was assessed using the *t*-test. Moreover, the predictive value of ultrasonography was evaluated.

The predictive value was estimated based on the classification results, which are a trade-off between the desired fraction of true positives (nonresponding classified as nonresponding) and the accepted fraction of false positives (responding classified as nonresponding). The area under the curve (AUC) is a measure of the predictive power and is defined as 1.0 for a perfect classifier, 0.5 for a random classification, and 0 for a completely incorrect classification.

## SUPPLEMENTARY FIGURE


