# Supplementary material for: Thermal tomography for monitoring tumor response to neoadjuvant chemotherapy in women with locally advanced breast cancer
Source: Oncotarget. 2017 Mar 25;8(40):68974–83. doi: 10.18632/oncotarget.16569 (PMC5620312; doi:10.18632/oncotarget.16569)
Supplement: Supplementary file 1 [file oncotarget-08-68974-s001.pdf]

Thermal tomography for monitoring tumor response to neoadjuvant chemotherapy in women with locally advanced breast cancer

Supplementary Material

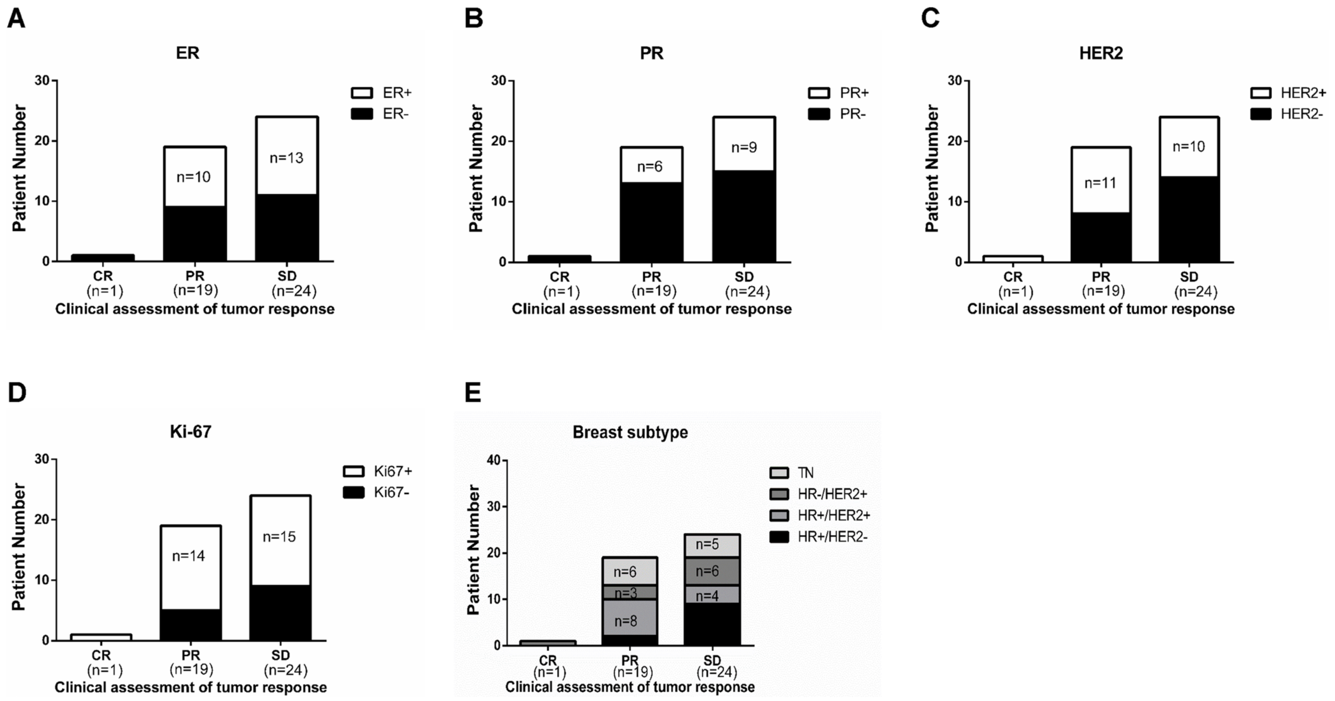

Supplementary Figure 1: The distribution of cancer-associated characteristics was classified following clinical assessment and REC of response measurements after six cycles of NAC.
